# Performance of quality improvement teams and associated factors in selected regional referral hospitals in Tanzania: a cross-sectional study

**DOI:** 10.11604/pamj.2021.38.223.23767

**Published:** 2021-02-26

**Authors:** Godfrey Kacholi, Albino Kalolo, Ozayr Haroon Mahomed

**Affiliations:** 1 Discipline of Public Health Medicine, University of KwaZulu-Natal, Durban, South Africa,; 2 Department of Health Systems Management, Mzumbe University, Mzumbe, Tanzania,; 3 Department of Public Health, St. Francis University College of Health and Allied Sciences, Ifakara, Tanzania

**Keywords:** Perceptions, quality improvement teams, regional referral hospitals

## Abstract

**Introduction:**

quality improvement teams facilitate improvement in the performance of the health facilities and simultaneously improving the quality of health services. There is scanty information on the factors associated with performance of quality improvement teams. This study aimed to assess the perceptions of members of the quality improvement teams on the factors influencing the performance of quality improvement teams in regional referral hospitals in Tanzania.

**Methods:**

a cross-sectional study was conducted in four regional referral hospitals in Tanzania. We used self-administered questionnaires to collect data from 61 members of quality improvement teams. Descriptive statistics were used to assess the perceived factors influencing team performance. Bivariate and multivariate logistic regression was used to test the association between perceptions of the team members and factors associated with team performance.

**Results:**

the overall mean perception score on team performance was high at 27.51 ± 4.62. Five factors namely: training (83.6%); communication (75.1%); team cohesiveness (71.5%); clarity of roles and responsibility (70.2%); team size and composition (65.5%); and self-assessment and learning (56.2%) were considered as the main drivers of team performance. Inadequate management support obtained the lowest score (36.1%). Multivariable regression analysis established a significant association between training, communication, clarity of roles and responsibilities, team size and composition, self-assessment and learning, management support and team performance.

**Conclusion:**

inadequate management support to the team was found to be a barrier to team performance. Managerial interventions should focus on provision of coaching and mentoring to the team while addressing resource challenges affecting the team performance.

## Introduction

Tanzania has been experiencing a “double burden” of communicable and non-communicable diseases (NCDs) [1]. Communicable diseases, perinatal, maternal and nutritional conditions accounted for almost 56% of all deaths followed by NCDs that caused about 33% of all death in 2016 in Tanzania [2]. Despite the several efforts to combat the “double burden” of diseases, such as establishment of a unit at the Ministry of Health, Community Development, Gender, Elderly and Children to steer formulation of NCD policies and guidelines the health outcomes have remained suboptimal in Tanzania [3, 4] characterized by a high maternal mortality ratio (556 deaths per 100,000 live births and an under-fives mortality rates stands at 54 deaths per 1,000 live in 2014 [5]. The life expectancy increased from 51 years in 2002 to 62 years in 2012 [6], partly due transformation of some communicable diseases such as Human immunodeficiency virus (HIV) resulting into chronic diseases as a result of massive roll-out of antiretroviral treatment (ART) [7]. Literature indicates that the double burden of disease has been negatively affecting the capacity of the health systems and hospital facility indicators [8]. Increased patient numbers has caused hospitals to experience immense challenges such as overcrowding, increased hospital stay, delayed referrals, uncoordinated health services and long patient waiting [9] whilst simultaneously causing staff workload to be extremely high [10]. As the results, the hospitals are facing the extreme challenges of providing quality care [9].

The Tanzania HealthCare Quality Improvement Framework [9] was re-launched in 2011, and implemented as a renewed strategy to improve provision of quality health services. The strategy focuses on institutionalizing the culture of continuous quality improvement at all levels of health care delivery by improving the working environment, strengthening leadership, commitment and coordination, strengthening relationships and trust among key stakeholders within and outside the health sector to achieve sustainable quality improvement programs at all levels of service provisions. The strategy recommends the use of Quality Improvement (QI) teams as an approach to ensure quality of services is improved and maintained at the health facility level. QI team consists of a small group of health professionals with skill-mix and expertise committed to achieve common goals for which they are collectively accountable [9].

The primary functions of the QI teams include strengthening and promotion of quality health service delivery in health facilities whilst simultaneously sustaining change by ensuring coordination and overseeing the designing, implementation and monitoring of all QI activities related to infrastructure, service delivery, commodities management and safety programmes at the health facility levels [9]. Positive impacts of QI teams in the health facilities in Tanzania have been reported in various studies [11, 12]. The reported impact of QI teams includes increased staff job satisfaction and reduced workload, relatively reduced patient waiting time, improved patient satisfaction and improved patients´ records management [13]. Despite the observed positive impacts of QI teams, and increase in the adoption and mainstreaming of the QI teams in the hospitals in Tanzania, a significant number of these teams have been performing below the expectations [14]. There has been heterogeneity in terms of teams´ activeness, effectiveness and consistency from one hospital to another [15].

Previous studies have reported various factors associated with team performance including socio-demographic factors such as age, level of education, working experiences [16]; communication, size of the team, management and leadership support, and availability of financial resources to support team activities [17-19]. However, studies on factors affecting the performance of QI teams in the hospitals in Tanzania are scarce - and this is the basis of our study. In this time where cost containment, performance and quality are emphasized, understanding factors that could influence team performance is fundamental to strengthen hospital QI teams. Therefore, the aim of this study was to assess the perceptions of members of the QI teams on the factors influencing the performance of QI teams in regional referral hospitals in Tanzania.

## Methods

**Study design and setting:** a cross-sectional study was conducted in four selected regional referral hospitals situated in four regions in Tanzania between April and August in 2018. The selected hospitals were Singida hospital located in Singida region, central Tanzania; Tanga regional referral hospital located in eastern part of the country; Mbeya hospital located in south-highlands of the country; and Sekou-Toure hospital located along the shore of Lake Victoria, north-western Tanzania.

**Study population and sampling:** a multi-stage sampling procedure was employed to select the hospitals. Firstly, all public-owned regional referral hospitals were categorized into two groups of high performing hospitals (with scores of 70% and above) and low performing hospitals (with scores of less than 40%) with regards QI implementation progress as recorded by the Ministry of Health and Social Welfare in 2016 [20]. Secondly, simple random sampling techniques using a lottery method was performed to obtain four hospitals—(Tanga and Singida hospitals) from the high performing category and (Sekou-Toure and Mbeya hospitals) from low performing category.. Thereafter, a convenience sampling method was used to enroll team members to participate in this study. This study targeted to recruit all seventy six members of QI teams across the four selected hospitals.

**Data collection tool and procedures:** a self-administered questionnaire was used to obtain data from members of QI teams. The development of the questionnaire was informed by the recommendation of previous studies that assessed team performances [17, 21]. The questionnaire consisted of two main parts. The first part of the questionnaire collected socio-demographic characteristics of study participants while second part of the questionnaire collected team-related factors. The questionnaire was reviewed by two experts in the field of hospital management to verify the contents, clarity and format of the questionnaire. The questionnaire was prepared in English language and then translated to Kiswahili language, the lingua franca in Tanzania. The questionnaire in Kiswahili version was back-translated into English version to maintain the accuracy and consistency of the questionnaire. The translation involved two stages. At first, the translation was done by the first author. The accuracy of translation was validated by two language experts. The questionnaire was pre-tested with 9 members of QI teams. The questionnaire was distributed to and collected from the recruited members of QI teams in their respective offices by the first author. Members of QI teams were requested to fill-in the questionnaire privately. After completion of filling in the questionnaires, the first author collected the questionnaires immediately.

### Variables and measurements

**Outcome variable:** the outcome variable was team performance. The outcome variable was obtained from the summation score of six items that were used to measure team performance. The items focused to assess the team in terms of: - (i) ability to address problems timely, (ii) level of support, (iii) implementation of QI activities, (iv) decision making, (v) achievement of set goals and (vi) accountability for the outcomes. Each item was measured in the form binary responses (yes/no). The maximum score attainable was 43. The mean and median scores were calculated. The mean score of ≥27 represents good performance and mean score of ≤27 represents weak performance.

**Independent variables:** there are two sets of independent variables namely: the socio-demographic variables and team-related variables. The socio-demographic variables consisted of gender (male or female) age (less than 35 years or more than 35 years); educational level (degree or none-degree holders); professionalism (clinical or none-clinical); length of service in the hospital (less than 10 years or more than 10 years), position in the team (leadership or ordinary member); and duration of membership in a team (less than 4 years or more than 4 years). Team-related variables were training, communication, team size and composition, roles and responsibilities, management support, self-assessment and learning and team cohesiveness. Each team-related variable was obtained after the summation of the self-reported items to obtain a composite score. Each item was assigned a yes/no response (additional file 1). From the composite score, we calculated the mean and mean percent scores. Variables with mean percent of above 50% were considered as important domains that could enhance team performance.

**Data processing and analysis:** the collected data was assessed for consistency and completeness on daily basis. Thereafter, the data was cleaned, coded and entered into the computer database using Microsoft Excel 2010. Then, data was exported to Statistical Package for Social Science (SPSS) version 16.0 for analysis. Descriptive statistics was used to determine frequencies and proportions for categorical variables while measures of central tendency were used for continuous data. Bivariate analysis followed by logistical regression was used to test for associations between dependent and independent variables. Backward stepwise regression analysis was performed to predict variables to be removed from the model. Results were considered significant for p-values less than 0.05.

**Ethical considerations:** approval to conduct this study was obtained from the National Institute for Medical Research, Tanzania (NIMR/HQ/R.8a/Vol.IX/2666) and from the Biomedical Research and Ethics Committee, University of KwaZulu-Natal in South Africa (BE: 606/17). Permission to conduct the study was also obtained from relevant authorities. Consents were sought from study participants after they were informed about the objectives, benefits and risks of the study.

## Results

**Characteristics of study participants:** a total of 61/76 members of QI teams participated in the study (response rate of 80.2 %). The mean age was 38.8 years (±7.46), with a median age of 40 (IQR: 33-43). Almost half (50.8%. n=31) of the study participants were female and had less than 10 years of service (67.2%, n=41) in the hospital. Seventy two percent (n=44) had less than four years of membership in the teams, 89% 88.5%, (n=54) were ordinary members of the teams and (68.9%, n=42) were neither members of the team nor members of hospital management teams of the total number of participants (Table 1).

**Table 1:** socio-demographic characteristics of study participants (n=61)

Socio-demographic factors	Frequency	Percent
**Gender**		
Male	30	49.2
Female	31	50.8
Total	61	100
**Age**		
Age < 40 years	20	32.8
Age > 40 years	41	67.2
Total	61	100
**Level of education**		
Non-degree graduates	8	13.1
Graduate with degrees	53	86.9
Total	61	100
**Professionalism**		
Non-clinical	34	55.7
Clinical	27	44.3
Total	61	100
**Length of service in the hospital**		
Less than 10 years	41	67.2
More than 10 years	20	32.8
Total	61	100
**Length of membership in the QI team**		
Less than 4 years	44	72.1
More than 4 years	17	27.9
Total	61	100
**Position in QI the team**		
Leadership	7	11.5
Ordinary member	54	88.5
Total	61	100

**Figure 1:**
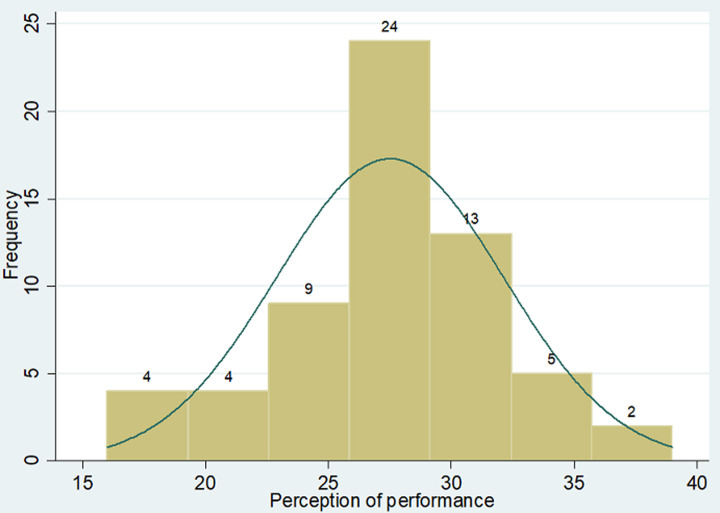
perception on team performance

**Perception on team performance:** the overall mean perception score on team performance was 27.51 ± 4.62 (78.6% of maximum score). The data was centrally located with the median score of 28 (IQR: 16-39) (Figure 1).

**Perceptions based on socio-demographic characteristics:** perceptions of team members based on their socio-demographic characteristics showed that female staff (n=17; 54.8%, p=0.143), ordinary team members (n=28; 52%, p=0.059), non-clinical professionals (n=19; 56%, p=0.361), members with less than 7 years (n=17; 53.1%, p=0.012) and those with less than 24 months of membership (n=22; 58%, p=1.194) had a more positive perception of the performance of the team. Team members with less than 40 years of age (n=18; 51.4%, p=0.498) and those with tertiary education (n=27; 50.1%, p=1.875) were less impressed by the performance of the team. Significant differences were found between the length of service (less than 7 years) (n=17; 53.1%, p=0.012) and position in the team (ordinary member) (n=28; 51.9%, p= 0.059) for the overall perception on the team performance (p<0.05). However, there was no significant difference in the overall perception on team performance across the other socio-demographic characteristics (Table 2).

**Table 2:** perceptions based on socio-demographic characteristics of study participants (n=61)

Characteristics	Performance above average		Performance below average		p-value
**Number**	**Percentage**	**Number**	**Percentage**
**Gender**					
Male	15	50.0	15	50.0	0.143
Female	17	54.8	14	45.2	
**Age**					
Age < 40 years	15	57.7	11	42.3	0.498
Age > 40 years	17	48.6	18	51.4	
**Education**					
Tertiary	26	49.1	27	50.1	1.875
Secondary and less	6	75.0	2	25.0	
**Position in team**					
Team leader	4	57.1	3	42.9	0.059
Ordinary member	28	51.9	26	48.1	
**Professionalism**					
Clinical	13	48.2	14	51.8	0.361
Non-clinical	19	55.9	15	44.1	
**Length of service**					
> 7 years	15	51.7	14	48.3	0.012
< 7 years	17	53.1	15	46.9	
**Length of membership**					
> 24 months	10	43.5	13	56.5	1.194
< 24 months	22	57.9	16	42.1	

**Figure 2:**
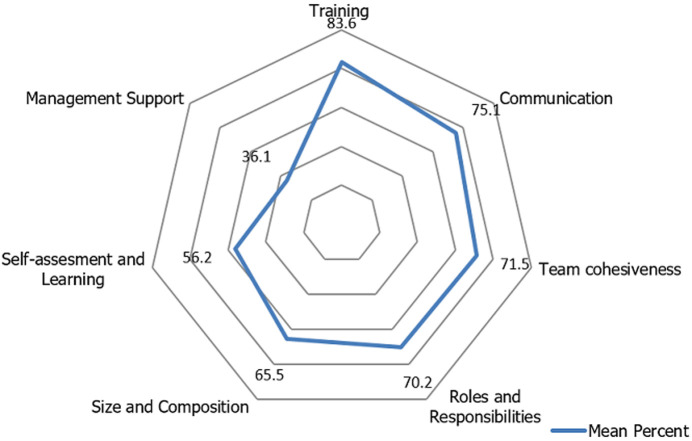
mean percent of perceptions on team-related factors

**Mean and mean percent score of team-related factors:** training was the highest scoring domain (83.6%; mean 5.02 ± 1.44) followed by communication (75.1%; mean 4.51 ± 1.26), clarity of roles and responsibility (70.2%; mean 4.21 ± 1.23) with the mean score of while management support obtained the lowest (36.1%; mean 2.16 ± 1.19) with the mean score of (Figure 2).

### Association of perceptions on performance of QI teams and associated factors

**Socio-demographic factors and perceptions on performance of QI teams:** bivariate and multivariate logistic regression analyses indicated that there was no statistical association between socio-demographic characteristics and perception on performance of QI teams. In multivariate analyses, gender (female) (OR 1.31; CI 0.45-3.83, p=0.617); age (< 40 years) (OR 1.83; 0.55-6.04, p=0.324); position (ordinary member) (OR 2.47: CI 0.43-14.19, p=0.311) and length of service (< 7 years) (OR 1.88; CI 0.52-6.73, p= 0.327) had an increased odds of positive perception on team performance. Education (tertiary) (OR 0.23: CI 0.04-1.48, p=0.121), professionalism (non-clinical) (OR 0.66 CI 0.22-1.99, p=0.458) and length of membership (OR 0.37: CI 0.11-1.32, p=0.127) showed a decrease in odds of positive perception towards team performance (Table 3).

**Table 3:** association of perceptions on performance of QI teams and socio-demographic factors (n=61)

Socio-demographic factors	Un-adjusted OR	95% CI	p-value	Adjusted OR	95% CI	p-value
						
**Gender**						
Female	1.21	0.39-3.74	0.705	1.31	0.45-3.83	0.617
**Age**						
< 40 years	1.44	0.46-4.55	0.481	1.83	0.55-6.04	0.324
**Educational level**						
Tertiary education	0.32	0.29-2.04	0.171	0.23	0.04-1.48	0.121
**Professionalism**						
Non-clinical	0.733	0.24-2.27	0.548	0.66	0.22-1.99	0.458
**Position**						
Ordinary members	1.23	0.18-9.23	0.792	2.47	0.43-14.19	0.311
**Length of service**						
< 7 years	0.95	0.31-2.91	0.912	1.88	0.52-6.73	0.327
**Length of membership**						
> 24 months	0.56	0.17-1.79	0.275	0.37	0.11–1.32	0.127

Level of statistical significance p<0.05

**Team-related factors and perceptions on performance of QI teams:** bivariate analysis indicated that communication (OR 3.12; CI 0.98-10.15, p=0.031); roles and responsibilities (OR 3.81; CI 1.00-16.03, p = 0.025), team size and composition (OR: 0.77; CI: 2.02-31.86, p< 0.0005), self-assessment and learning (OR 13.75; CI 3.32-65.75, p <0.0001), management support (OR 4.23; CI 1.15-17.45, p=0.0138), team cohesiveness (OR 9.02; CI 1.99-54.42, p =0.0007) were found to be significantly associated with positive perception of team performance. Although training (OR 1.72; CI 0.40-7.82, p =0.403) showed increased odds of a positive perception of team performance, the results were not statistically significant. After multivariable analysis, training (OR 156.09; CI 2.00-121.61, p=0.023), communication (OR 9.18; 1.09-77.67, p=0.042), roles and responsibilities (OR 31.81; CI 1.39-726.57, p=0.030), team size and composition (OR 12.38; CI 1.14-134.37, p=0.039), self-assessment and learning (OR 31.64; CI 1.93-516.76, p=0.015), and management support (OR 25.29; CI 1.43-446.63, p=0.027) were found to be predictive factors for positive perception of team performance. Although team cohesiveness (OR 13.52; CI 0.82-223.53, p=0.069) showed increased odds of a positive perception of team performance, the results were not statistically significant (Table 4). The wide confidence intervals in bivariate and multivariate analysis are indicative of small sample size, sparse frequency within the variables as well as interaction between the various factors and must be interpreted with caution. The backward stepwise regression analysis showed that there was hyperinflation of the odds on roles and responsibilities of the team, training, team size and composition, management support, team cohesiveness, and self-assessment and learning.

**Table 4:** association of team-related factors and perceptions on team performance (n=61)

Domains	Un-adjusted OR	95% CI	p-value	Adjusted OR	95% CI	p-value
Training	1.72	0.40-7.82	0.403	156.09	2.00-121.61	0.023**
Communication	3.12	0.98-10.15	0.031*	9.18	1.09-77.67	0.042**
Roles and responsibilities	3.81	1.00-16.03	0.025*	31.81	1.39-726.57	0.030**
Team size and composition	0.77	2.02-31.86	0.0005*	12.38	1.14-134.37	0.039**
Self-assessment and learning	13.75	3.32-65.75	0.0001*	31.64	1.93-516.76	0.015**
Management support	4.23	1.15-17.45	0.0138**	25.29	1.43-446.63	0.027**
Team cohesiveness	9.02	1.99-54.42	0.0007*	13.52	0.82-223.53	0.069**

* = level of statistical significance p<0.1; **= level of statistical significance p<0.05

## Discussion

Globally, hospitals strive to improve the quality of health services and Tanzania is no different. The use of the teams provides the hospital management with evidence-based measures and recommendations required to improve and sustain quality of care. The overall mean perception score on team performance was high at 27.51 ± 4.62. This result suggests the perceptions of the team members towards the performance of their teams´ were predominantly positive. These results are consistent to results of the cross-sectional study conducted in two mission referral hospitals in Kenya that found that staff had positive perception on team performance [22]. Positive perceptions could be attributed to continuous trainings and technical support provided to the teams that increases team motivation during the implementation of team activities [23].

**Socio-demographic factors and perceptions on performance of QI teams:** the finding showed that positive perception was higher among female participants versus male participants (OR 1.31; CI 0.45-3.83, p=0.617). This result is contrary to the results of the study that examined the influence team composition and gender-based cues on perceptions of team performance that found that women were less impressed by the performance of the team [18]. The results of the study may be attributed to the dramatically gender-skewedness of the Tanzania health workforce, with overwhelming majority being female [24]. Similarly, a cross-sectional study conducted at Stanger Hospital in South Africa found that the majority of the staff were women, and therefore gender composition could influence perception of staff [25].

This study did not establish any significant association between perception on the team performance and age (OR 1.83; 0.55-6.04, p=0.324). These results are inconsistent to results of the study conducted within 42 health facilities in South Africa that found that staff with above 51 years of age had positive perceptions towards QI interventions [26]. Some studies have shown that staff with younger age tend to be active, innovative and fast learners, which could affect their perceptions [27]. Multivariable analysis established that education (tertiary) showed a decrease in odds of perception towards team performance (OR 0.23: CI 0.04-1.48, p=0.121). Contrary to the results of the current study, literature has shown that individuals with a more advanced level of education are more likely to have positive perception than those with low level of education [28]. Positive perception of highly educated team members may be due to their capacity to transform the acquired knowledge and skills into practices.

Although there was no significant association between professionalism of team members and team performance (OR 0.66; CI 0.22-1.99, p= 0.458), descriptive results indicated that majority (55.9%) of team members with non-clinical professionalism were impressed by the performance of the team. This inference is inconsistent with the results of a study conducted in Canada that found that health professionals in clinical category especially nurses were impressed by the performance of inter-professional healthcare teams [29]. However, it is important to underscore that the interactions of clinical and non-clinical support staff have the powerful influence on the activeness and functioning of the QI teams. Our study did not find significant association between the length of membership (less than 24 months) performance of the team (OR 0.37; CI 0.11-1.32, p=0.127). Although the literature regarding the association between team performance and duration of membership in the team in the healthcare context is limited, existing evidence on team cohesiveness showed no significance difference between length of services and team effectiveness [30].

**Team-related factors and perceptions on performance of QI teams:** training of the QI teams was found to be an important factor influencing team performance (OR 156.09; CI 2.00-121.61, p=0.023). This result is corroborated by results from a study conducted in the selected regional referral hospitals in Tanzania showed that training contributed to the performance and sustainability of the QI teams [12]. Continuous training provides team members with opportunities to improve their skills and competences that could enable the team to perform optimally [30]. In the current study, it appears that majority of the team members attended trainings related to patient care, teamwork and team building, patients and staff safety and application of various QI tools. This suggests that team members who benefited from these trainings were most likely to have enthusiasm to ensure QI efforts are realized. The outputs of these trainings could improve team collaborations [31]; and more likely to enable team members to alternate roles, resulting into improved team performance.

Communication appears to be an important factor that influenced the performance of the QI teams (OR 9.18; CI 1.09-77.67, p=0.042). This result is consistent with the results of a study conducted in Kenya which found that the effectiveness of the hospital-based quality teams was attributed to the effective team communication [22]. It appears that the teams had well-designed communication mechanisms for information sharing within the team, with staff and hospital management. Functional communication mechanisms underscore the interplay between effective team communication and improved team interactions [31]. In the current study, it is perceived that team benefited from the mutual respect and understanding amongst members of the team. This could be due to presence of effective team leadership whose major role is to ensure that teams plans and functions are well-coordinated and communicated within the team and with hospital management and staff.

This study found significant association between clarity of clarity of roles and responsibilities and perceptions on team performance (OR 31.81; CI 1.39-726.57, p=0.030). This result is supported by the results of a systematic review of articles published between 2008 and 2018 that found clarity of roles and functions of the team offered an impetus to the overall team´s effectiveness [32]. Results of the current study suggest that roles of the team were well-known by overwhelming majority of the members, resulting into their understanding of routine team functions. This appears to be true because through continuous training, team members are more likely to be reminded about why their teams were formed. Studies have shown that role clarity of the team eliminates considerably potential duplication of work and unnecessary team conflicts [19, 33].

The current study established significant association between team size and composition and perceptions on team performance (OR 12.38; CI 1.14-134.37, p=0.039). This may be attributed to the nature of team formation as it consists of members with skill diversities and experiences. The literature indicates that teams with disciplinary composition are effective due to diversity of its members in terms of their professional career path, skills, knowledge and experiences that brings richness in team performance [19]. The results of the current study suggest that the heterogeneity of team played pivotal role in creating practical solutions to problems affecting the team and quality of care [34]. In addition, the current size (small team) was cited by members of the teams as appropriate in overseeing the implementation of various QI interventions at the hospital level. This insight could be due to the easiness when it comes to team mobilisation and management [33]. Small teams are also viewed as less conflict-prone groups. However, previous studies have reported that team performance is much evident in larger teams as compared to smaller teams [35, 36].

Both bivariate analysis (OR 13.75; CI3.32-65.75, p<0.0001) and multivariable analysis (OR 31.64; CI1.93-516.76, p=0.015) established significant association between team´s self-assessment and learning and perceptions on team performance. Teams that regularly focuses on assessing and learning on how they can perform better and better were predicted to be effective [37]. The results of the current study suggest that QI teams were adequately monitoring their progress from time to time to detect any mistakes resulting from new developments, practices and procedures from within and outside the hospital. Self-assessment is important as it helps to identify strengths and challenges of the team and enable the team to develop strategic interventions to overcome the challenges for better performance [38]. Self-assessment and learning remain an important process for team performance that should be embraced by both team leadership and the hospital management.

Although multivariable analysis showed that hospital management support was significantly associated with the perceptions on team performance (OR 25.29; CI1.43-446.63, p< 0.027, results from descriptive statistics showed that the support of hospital management to the team obtained the lowest mean percent score (36.1%; mean 2.16 ±1.19). These results are corroborate by the results conducted in the selected hospitals in Tanzania that concluded that inadequate management support to the teams affected negatively to the performance and sustainability of the teams [12]. Previous studies suggests that less optimal support of the organisational management during the change process affects adversely to the performance of any intervention [39]. The formation of QI teams in Tanzania is often guided by volunteerism of hospital staff to join the team. As a result, membership in the team is being perceived as an added workload. The inclusion of members from hospital management appeared to have no substantial contribution to the team performance. This is possibly because the hospital managements could not disentangle what constituted the roles of the QI teams and that of the hospital management teams. This oversight may potentially lead to the management to allocate inadequate resources to the team, mistrust and communication breakdown which may strain the relationship between the team and management [33].

**Strengths and limitations:** the strengths of this study is that this is amongst the first study to systematically assess the perceptions of members of the QI teams on the factors influencing the performance of QI teams in regional referral hospitals in Tanzania. The study covered several hospitals, and explored multiple factors that can potentially influence team performance. Limitations of our study are that the findings of this study might not be generalizable because the sample size is relatively small; however, the findings can be transferred. Moreover, examined factors were not exhaustive, and we did not collect qualitative data on factors influencing team performance to help us to provide in-depth information on issues that came out in the quantitative results.

## Conclusion

The study identified training, communication, team size and composition, clarity of roles and responsibilities of the team, team cohesiveness, and teams´ self-assessment and learning were potential factors that influenced the performance of the QI teams. Inadequate hospital management support to the team was found to be a barrier and had a negative impact on the performance of the teams. Managerial interventions should focus on provision of mentoring and coaching through continuous supportive supervision and addressing resource challenges that have the potential to influence performance of the teams. Interventions for improving the performance of the QI teams should also consider the influence of socio-demographic parameters such as education, professionalism and length of membership.

### What is known about this topic

- Quality improvement teams are formed with hospital staff from middle and top management. These teams were established in hospitals to speed of decision-making and increase commitment for quality improvement;
- An effective team is usually built on the following dimensions; clear direction, defined roles, appropriate communication, strong management support, mutual respect and accountability, team formation, time management, and ability to solve problems.

### What this study adds

- Training is an important component of team performance;
- Positive perceptions on the team performance reflect a belief that QI teams are important arms of the hospital that should supported, developed and sustained by the hospital management.
